# Effects of Fragmentation and Sea-Level Changes upon Frog Communities of Land-Bridge Islands off the Southeastern Coast of Brazil

**DOI:** 10.1371/journal.pone.0103522

**Published:** 2014-07-28

**Authors:** Gabriela B. Bittencourt-Silva, Hélio R. Silva

**Affiliations:** Departamento de Biologia Animal, Universidade Federal Rural do Rio de Janeiro, Seropédica, Rio de Janeiro, Brazil; University of Kent, United Kingdom

## Abstract

We investigate the composition of anuran communities of land-bridge islands off the southeastern coast of Brazil. These islands provide natural long-term experiments on the effects of fragmentation in the Brazilian Atlantic Forest (BAF). We hypothesize that Pleistocene sea-level changes, in combination with other abiotic variables such as area and habitat diversity, has affected anuran species richness and community composition on these islands. Data from the literature and collections databases were used to produce species lists for eight land-bridge islands and for the mainland adjacent to the islands. We assess the effects of area, number of breeding habitats and distance to the mainland upon anuran species richness on land-bridge islands. Additionally we use nestedness analysis to quantify the extent to which the species on smaller and less habitat-diverse islands correspond to subsets of those on larger and more diverse ones. We found that area has both direct and indirect effects on anuran species richness on land-bridge islands, irrespective of distance to the mainland. However, on islands with comparable sizes, differences in species richness can be attributed to the number and quality of breeding habitats. Anuran communities on these islands display a nested pattern, possibly caused by selective extinction related to habitat loss. Common lowland pond-breeders were conspicuous by their absence. In the BAF, the conservation of fragments with a high diversity of breeding habitats could compensate for the generally negative effect of small area upon species richness. We suggest that sea-level changes have an important role in shaping composition of anuran species on coastal communities.

## Introduction

Fragmentation is a potential causal factor affecting species distributions. Around the world, especially in the tropics, forests are being reduced to fragments to make space for agriculture, industry and urban development. The Brazilian Atlantic Forest (BAF) has been severely fragmented since colonization in the sixteenth century, leading to the devastation of approximately ninety percent of its original distribution [1]. The amazingly rich diversity of plants and animals of this ecoregion has been seriously threatened by the fragmentation process [2]. Understanding the impact of habitat fragmentation on species populations is crucial to mitigating its damage and delineating strategies for conservation.

According to Watson [3], there are two broad classes of patchy habitats: ‘islands’ are patches that have always been isolated, whereas ‘fragments’ are patches that were previously connected. In contrast to most oceanic islands, land-bridge islands are fragments previously connected to the mainland. As such, they provide natural experiments on the effects of fragmentation upon terrestrial and fresh water animals and plants over potentially longer timescales than those associated with human mediated changes. In general, species richness tends to increase with increasing island area, which can affect both population size and diversity of habitats. In other words, area has an effect on extinction rate and species diversity, respectively [4]. Because area and habitat diversity are usually correlated [5], [6] it is difficult to determine which one better predicts species richness and opinions on this are divided [6]–[8].

Given that they are mostly poor dispersers across salt-water barriers, amphibians on land-bridge islands provide excellent, though under exploited, opportunities to investigate the effects of fragmentation upon community structure. In Brazil, throughout the coast of Rio de Janeiro and São Paulo states, there are hundreds of land-bridge islands that have been isolated since the Holocene. Some of these islands harbour diverse communities of frogs, including some endemic species [9]–[12]. Several studies with different taxonomic groups have demonstrated that insular communities are not randomly distributed; rather, they tend to be structured in a nested pattern [13]–[15]. Communities are said to be nested when faunas of depauperate sites constitute non-random subsets of those of richer sites. [16]. For instance, if islands of a nested archipelago are arranged by species richness, the ones with fewer species should have subsets of those found on richer islands.

Considering the process of island formation and the relationship between anuran species and habitat characteristics, we hypothesize that a combination of physical variables, acting synergistically, regulates anuran species richness on land-bridge islands irrespective of the distance to the mainland. The communities of extant insular frog faunas off the coast of Rio de Janeiro and São Paulo provide opportunities to test this hypothesis and to contribute insights to the debate as to the relative importance of historical events, area and habitat diversity for species richness. Our goal is to assess the effects of habitat fragmentation on anuran communities using land-bridge islands as long-term natural experiments. This is the first study comparing communities of frogs from islands off the Brazilian coast.

## Materials and Methods

### Study area

**Figure 1:**
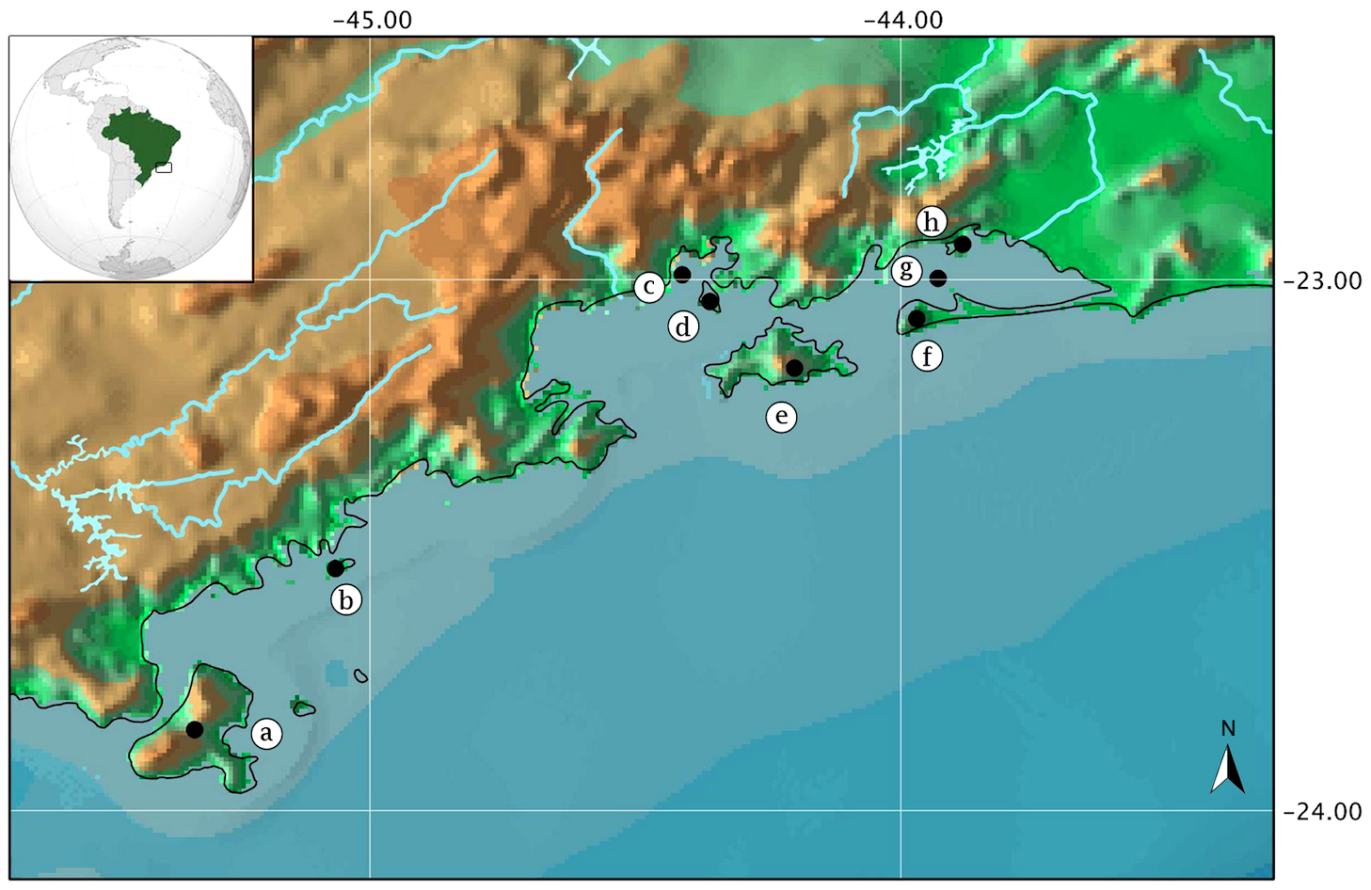
Map showing the localities of the eight land-bridge islands included in this study. Ilha de São Sebastião (a), Ilha Anchieta (b), Ilha de Itanhangá(c), Ilha da Gipóia (d), Ilha Grande (e), Ilha da Marambaia (f), Ilha de Jaguanum (g), Ilha de Itacuruçá (h).

The study region is located in the coastal southeast of Brazil, between latitudes 22°45′ and 24°30′ South, and longitudes 43°30′ and 45°30′ West (Figure 1). Table 1 presents the area, maximum altitude, minimum distance to mainland and other features of the eight islands included in this study. This region is characterized by the abrupt transition between the narrow coastal plane and the steep slopes of Serra do Mar (a mountain chain that extends beyond the study area: from the State of Espírito Santo in the North to the State of Santa Catarina in the South) [1]. Just a few kilometres off the coast there are hundreds of islands and islets. These were isolated from the mainland by rising sea levels most recently about 10,000 years before present (10 kyr bp) at the end of the Last Glacial Maximum [17]. The occurrence of submerged paleoriver canals along the coast provides clear evidence of previously lower sea levels [18]. Vermetid gastropods and oyster shells found hundreds of meters inland, away from the present coastline of the states of São Paulo and Rio de Janeiro, provide evidence that *c.* 5 kyr bp sea level was 3.5±1 m higher than at present [19], [20]. The associated transgressive events would have substantially reduced the extent of coastal lowland areas, especially on the islands. The islands included in the present study have been repeatedly isolated from (and reconnected with) the mainland in the past 500 kyr bp [21]. Hence, the fauna and flora of these islands are expected to be relicts of mainland populations. Ilha da Marambaia represents a special case. As shown on the map (Figure 1), this island is connected to the east with the mainland by a tombolo 42 km long formed about 5 kyr bp [22].

**Table 1:** Description of the eight islands included in the present study.

Island	Latitude	Longitude	Size	SM	Species	Area (km^2^)	Elev (m)	Dist (km)	MAP (mm)	MinT (°C)	MaxT (°C)
São Sebastião	−23.847750	−45.330173	L	1, 2, 3	35	348,3	1379	1,9	1,500	20.3/12.7	26.6/33.2
Grande	−23.166667	−44.200000	L	1, 2	31	193,0	1031	3,0	1,977	19.8/17.1	27.0/30.4
Marambaia	−23.073298	−43.969865	M	1, 2	26	42,0	647	9,5	<1,200	19.6/06.7	28.5/40.4
Itacuruçá	−22.933333	−43.883333	S	1, 2	13	9,9	335	0,2	NA	NA	NA
Anchieta	−23.544310	−45.064886	S	1, 2	18	8,3	339	0,5	NA	NA	NA
Gipóia	−23.041315	−44.359547	S	1	7	6,0	286	0,7	NA	NA	NA
Jaguanum	−22.997582	−43.929731	S	1, 2	4	2,5	232	6,6	NA	NA	NA
Itanhangá	−22.990833	−44.411667	VS	1	6	0,3	79	1,3	NA	NA	NA

Island size: large (L), medium (M), small (S) and very small (VS). Sampling methods (SM): visual encounter (1), pit-fall traps (2) and plots (3). Maximum elevation (Elev), minimum distance to mainland (Dist), mean annual precipitation (MAP), mean minimum temperature (MinT)/absolute minimum, mean maximum temperature (MaxT)/absolute maximum. Data not available (NA).

Our study region is included in the Atlantic Forest Biome [23] and the main vegetation type is classified as Dense Ombrophilous Forest [24], although some islands also have both restingas (sand dune shrub and forested areas) and open areas with rocky outcrops covered with bromeliads. According to the classification of Köppen, the climate of the region is tropical monsoon (Am). Table 1 shows climatic parameters available for the study islands. The mean annual temperature is between 23° and 25°C, with the higher temperatures occurring between December and February, and the lower between June and August [25], [26]. On the largest island, Ilha de São Sebastião, the mean annual precipitation is ca 1500 mm, with high levels of precipitation (ca 200 mm/month) occurring between December and March, and ca 90 mm/month from April to November [25]. On the next two largest islands, Ilha Grande and Ilha da Marambaia, the mean annual precipitation is ca 1900 mm and 1200 mm, respectively [26], [27]. The maximum altitudes of the islands range from 79 m on Ilha de Itanhangá (the smallest of the islands) up to 1379 m on Ilha de São Sebastião.

The study islands vary in the availability of temporary and permanent water bodies. For instance, only the two largest islands (Ilha de São Sebastião and Ilha Grande) have a complex drainage system, with both rivers and streams. Streams and rivulets can be found on all but two islands. Only temporary ponds are present on Ilha de Itanhangá and temporary ponds and streams on Ilha de Jaguanum. Although there are hundreds of islands in the study region, only a few have been surveyed and have published species lists.

Human occupations on these islands date back to the early 16^th^ century and on the largest, is marked by various interventions such as sugar cane and coffee cultivation, prison and quarantine station for slaves [28]–[31]. Currently, most of these islands are partially protected as state or federal conservation units.

### Database

We compiled records of anuran species from eight islands and seven adjacent localities on the mainland (Figure 1, Table S1) based on the literature and online herpetological collections databases available up until June 2013 (Table S2). We used the taxonomic classification of Frost [32]. Cicchi *et al.* [33] identified at least two distinct and still undescribed species of *Adenomera* (*Leptodactylus marmoratus* group) on Ilha Anchieta. Similarly, on Ilha de São Sebastião there are records of three undescribed species, two of the *Ischnocnema lactea* species series and one species of the *Scinax catharinae* group (F. Centeno pers.com.). Additionally, one adult female specimen of *Gastrotheca* was reported by Izecksohn and Carvalho-e-Silva [34]. On six islands (Table S1) there are members of the *Scinax perpusillus* group thought to represent species yet to be described [11], [33]. The only *Flectonotus* found on Ilha Grande is a juvenile and could not be identified. Table 1 shows the sampling methods used on each island according to the literature. As a result of the difference in methods applied, survey effort cannot be compared among islands.

### Breeding habitat classification

We used data from the literature [35]–[38] to classify species breeding habitats according to their reproductive modes (RMs), using the classification proposed by Haddad and Prado [39]. For species with multiple RMs, only the primary ones [39] were considered for the analyses. Table 2 provides brief descriptions of macro-habitats and breeding habitats, and their distributions on the islands are presented in Table 3. A list of RMs adapted from [39] is presented in Table S3.

**Table 2:** Brief description of the macro-habitats and breeding habitats used by anurans on the islands.

Category		Description
Macro-habitats	Forest	Humid, dense, heterogenic and perennial forest;
	Open area	Rocky shores and plain areas or lowlands without dense vegetation coverage;
	Restinga	Open physiognomy, sandy soil, scrub vegetation and frequent presence of bromeliads;
Breeding habitats	Bromeliad	Bromeliads on trees, over rocks or on ground;
	Canopy	Above ground portion of forest formed by tree crowns;
	Stream	Small lotic water bodies (creek, stream or rivulet);
	Film of water	Rocks with thin film of running water (isolated, near rivers, streams or waterfalls);
	Leaf litter	Layer of decomposing leaves, trunks and twigs on forest floor;
	Pond	Temporary or permanent lentic water bodies;

**Table 3:** Macro-habitats and breeding habitats recorded on each island.

Macro-habitat	Breeding habitat	SAO	GRD	MAR	ITA	ANC	GIP	JAG	ITN	Total
Open Area	Bromeliad	+	+	+	+	+	+	+	+	**8**
	Film of water	+	+	+	+	+	+	+	+	**8**
	Pond	+	+	+	+	+	+			**6**
	Creek	+	+	+	+	+				**5**
Forest	Bromeliad	+	+	+	+	+	+			**8**
	Film of water	+	+	+	+	+	+	+	+	**8**
	Leaf litter	+	+	+	+	+	+	+	+	**8**
	Pond	+	+	+	+	+	+	+	+	**8**
	Stream	+	+	+	+	+	+	+		**7**
	River	+	+							**2**
	Canopy	+	+	+	+	+	+	+	+	**8**
Restinga	Bromeliad		*	*		*				**3**
	Pond		*	*		*				**3**
**Total**		**11**	**13**	**12**	**10**	**12**	**9**	**7**	**6**	

Ilha de São Sebastião (SAO), Ilha Grande (GRD), Ilha da Marambaia (MAR), Ilha Anchieta (ANC), Ilha de Itacuruçá (ITA), Ilha da Gipóia (GIP), Ilha de Jaguanum (JAG), Ilha de Itanhangá (ITN). Macro-habitat formed after the isolation process and therefore not included in the analyses (*).

### Data analysis

#### Species richness

**Figure 2:**
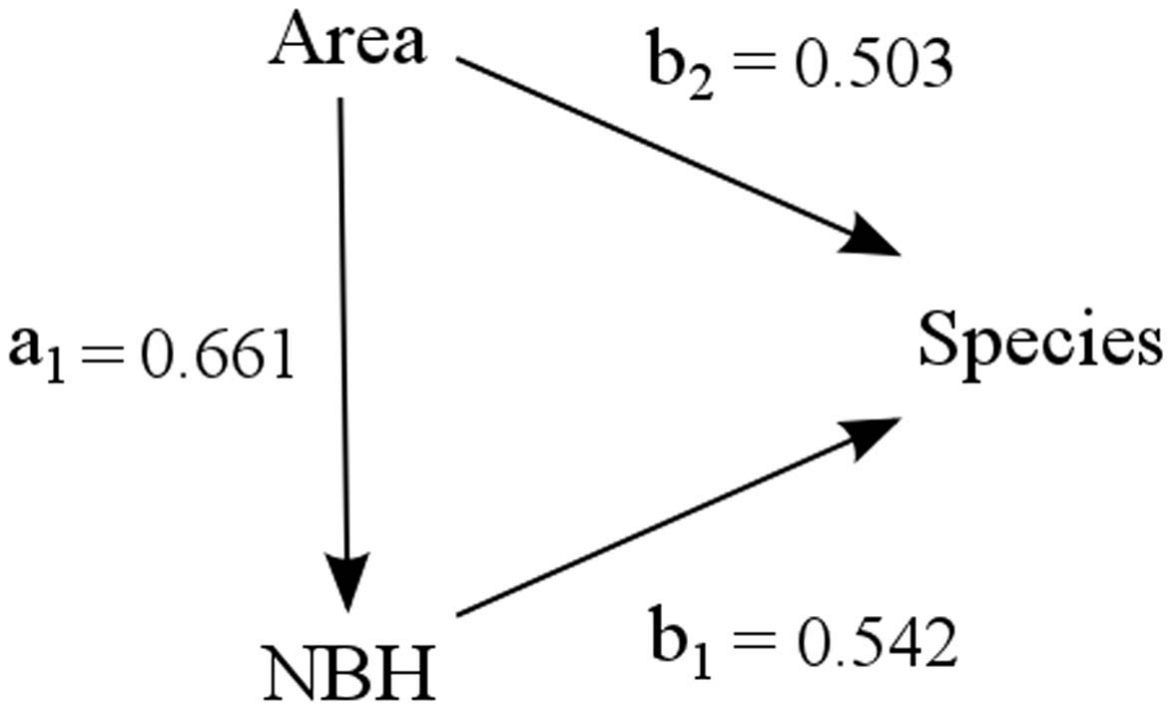
Path model of species number as a function of area and number of breeding habitats. Arrows indicate the direct effect of one variable on another. Number of suitable breeding habitats (NBH). Path coefficients represent: (a1) direct effect of area on NBH; (b1) direct effect of NBH on species number; and (b2) direct effect of area on species number.

We used univariate linear regression to analyse the relationship between species richness and (1) area, (2) number of suitable breeding habitats (NBH) and (3) minimum distance to the mainland, in order to evaluate the importance of these three variables in regulating the number of anuran species on the islands. We also performed a multiple regression of species richness as a function of area and NBH. Using the results from the previous analyses, we conducted a path analysis [40], which uses an a priori model, to evaluate the direct and indirect effects of causal variables, assuming linear relations among them – an example can be seen in Kohn and Walsh's study [41]. Our hypothesis assumes that area has a direct effect on NBH, and that both variables affect directly the number of species on the islands. Hence, area has a direct and an indirect effect on the species richness. Figure 2 shows the path model, representing the proposed relationships among the variables.

The occurrence of some restinga-dwelling species (*Aparasphenodon brunoi*, *Rhinella pygmaea* and *Xenohyla truncata*) on Ilha da Marambaia possibly resulted from dispersal through the tombolo (macro-habitat: restinga) after the formation of the island. Thus, inclusion of these species could confound the analysis and, for this reason, we conducted the analyses excluding the breeding habitats associated with restinga (and its respective species). The data analysed are shown in Table 1. The analyses were carried out using the R environment (version 2.15.2) [42] and the MASS package (version 7.3-22) [43].

#### Species composition

To quantify nestedness in the composition of species communities on the study islands we used the NODF metric [44], which ranges from zero (no nestedness) to 100 (perfectly nested). NODF is calculated from a presence-absence matrix with species (rows) versus islands (columns). Nestedness can be quantified between pairs of rows, and pairs of columns, independently, allowing the evaluation of species composition and species occurrence [44]. Herein we are concerned with nestedness in species composition as determined through comparisons of columns in the presence-absence matrix. Species were sorted from top to bottom in decreasing order according to their frequency, and islands were sorted from left to right in decreasing order according to NBH (not including restinga). If the islands have the same NBHs, the island with larger area is placed on the left. To test the statistical significance of the observed pattern (i.e. if it is more strongly nested than expected by chance alone), we used a null model that randomly reassigns species to islands while maintaining the observed numbers of species per island. The analyses were conducted using the NODF-Program [45].

## Results

### Species richness

The results show that distance to the mainland has no relationship with the number of species on the islands. In contrast, there is a strong positive correlation between number of anuran species per island (i.e. species richness) and area (Pearson's r^2^ = 0.74; *F*_16_ = 17.2; *P*<0.01) and between species richness and NBH (Pearson's r^2^ = 0.76; *F*_16_ = 19.5; *P*<0.01). Multiple regression of number of species as a function of area and NBH shows that, together, these variables explain 91% of the species richness on the islands (adjusted r^2^ = 0.91; *F*_25_ = 24.3; *P*<0.01; *P*_area_ and *P*_NBH_<0.05). The small *P* value in the multiple regression is due to the positive correlation between area and NBH (Pearson's r^2^ = 0.44; *F*_16_ = 4.6; *P*<0.01).

Based on these above results, we conclude that area and NBH are not acting independently upon the number of species on the islands, but it is not clear how much each of the variables contributes to species richness. Table 4 summarizes the results of the direct, indirect and total coefficients of species number as a function of area and number of suitable breeding habitats used by anurans (NBH) on the islands. Direct effects are represented by the path coefficients a_1_, b_1_, and b_2_. The first path coefficient is the simple regression coefficient for the standardized variables area and NBH. The other two (b_1_, and b_2_) are standardized partial regression coefficients from the multiple regression of species per island as a function of area and NBH. The path coefficient for the indirect effect of area is calculated by multiplying the direct effects of area and NBH on the species number (i.e. a_1_b_1_). The total effect of area upon species richness is the sum of the direct (b_2_) and the indirect (a_1_b_1_) effects of area on species number. The total effect of NBH is the direct effect of this variable on the number of species (b_1_). The direct effect of NBH on species number is similar to the direct effect of area on species number, but the sum of both, direct and indirect effects of area indicates that this variable has a stronger contribution on the number of species on the islands.

**Table 4:** Path and effect coefficients of species number as a function of area and number of suitable breeding habitats used by anurans (NBH) on land-bridge islands off the southeastern coast of Brazil.

Coefficient		Effect	Results
Path coefficient	Area on NBH	Direct	a_1_ = 0.661
	NBH on species number	Direct	b_1_ = 0.542
	Area on species number	Direct	b_2_ = 0.503
	Area on species number	Indirect	(a_1_b_1_) = 0.358
Effect coefficient	Area on species number	Total	b_2_+(a_1_b_1_) = 0.861
	NBH on species number	Total	b_1_ = 0.542

### Species composition

We found records of 63 species of anurans, distributed in 30 genera and 11 families, among the eight islands, and 105 species, distributed in 36 genera and 13 families, in the mainland localities (Table S1). Cycloramphidae and Leptodactylidae occur on all islands, whilst Aromobatidae and Ceratophrydae are only present on the mainland. Hylidae is the most speciose family both on the islands and in the mainland, accounting for 37% and 49% of the species, respectively. Forty-eight of the 63 species recorded for the islands are also found on the adjacent mainland localities. Of the remaining species, six occur in other mainland localities not included in this study, six are not assigned to named species (see Table S1), and three are endemic (*Hylodes fredi* and *Proceratophrys tupinamba* to Ilha Grande and *Leptodactylus marambaiae* to Ilha da Marambaia). The genera *Adenomera* (*A. marmorata*) and *Thoropa* (*T. miliaris* and *T. taophora*) occur on all islands. *Hypsiboas*, *Scinax* and species of the *Scinax perpusillus* group are absent only from Ilha de Jaguanum (Table S1). It is notable that some species that are widely distributed and abundant on the mainland are not present on the islands. For instance, only three of the 10 species of *Dendropsophus* listed for the adjacent mainland localities occur on the islands. Similarly, *Scinax similis* and *Leptodactylus fuscus* are not reported to occur on the study islands. All these species are associated with forest edges or open lowland areas.

The result of the nestedness analysis shows a highly nested pattern for species composition (NODF = 70.1), indicating that, as expected, anuran species are non-randomly distributed on the islands (*P*<0.001, for both null models). Because of the strong correlation between area, altitude and NBH, the same matrix order is obtained when sorting the islands according to each of these variables.

### Diversity of reproductive modes

**Figure 3:**
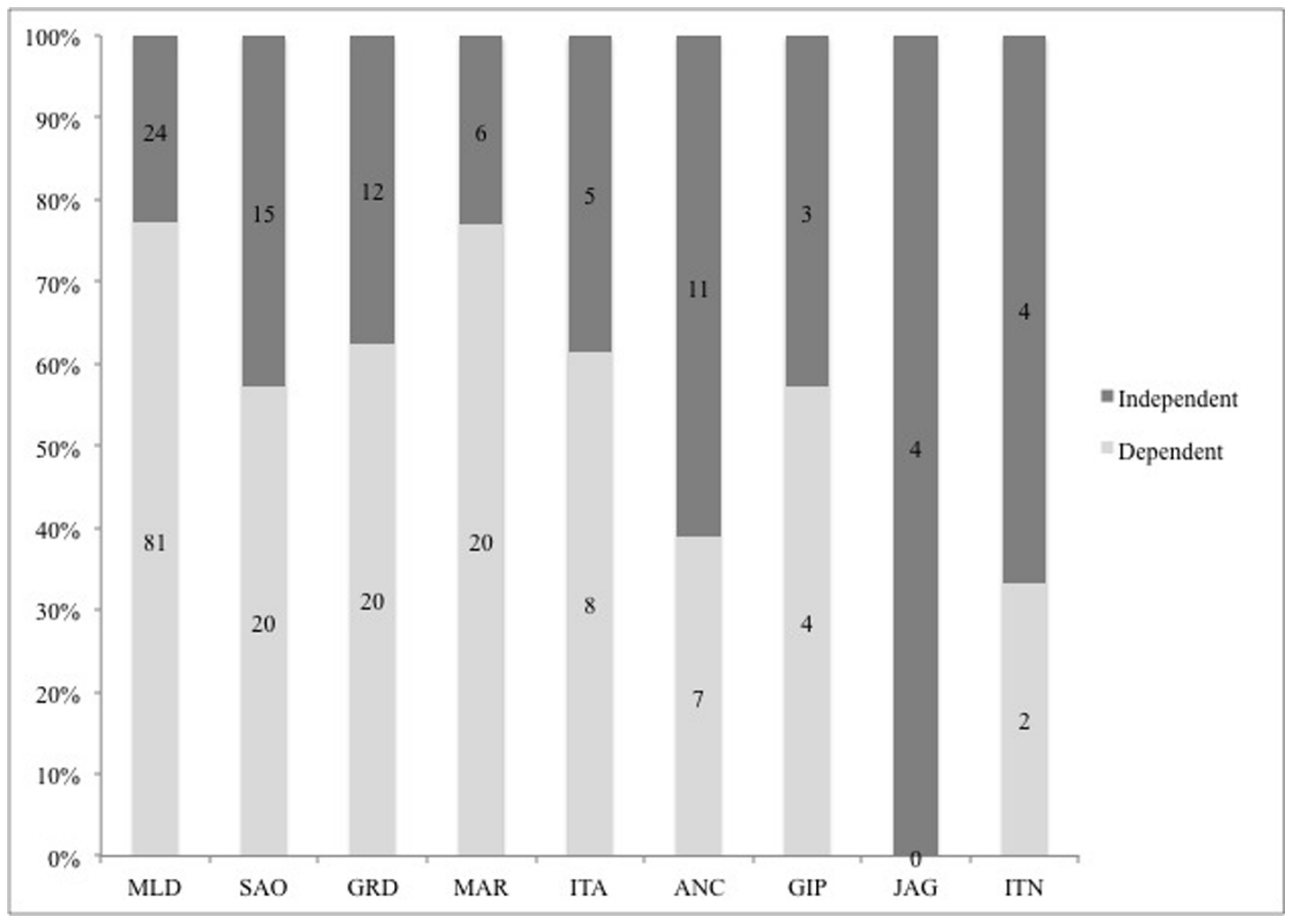
Percentage of frog species dependent and independent of water bodies for reproduction. Mainland (MLD), Ilha de São Sebastião (SAO), Ilha Grande (GRD), Ilha da Marambaia (MAR), Ilha Anchieta (ANC), Ilha de Itacuruçá (ITA), Ilha da Gipóia (GIP), Ilha de Jaguanum (JAG), Ilha de Itanhangá (ITN). Numbers on the bars indicate the actual number of species, and numbers in parentheses represent the area of the islands in ha.

**Figure 4:**
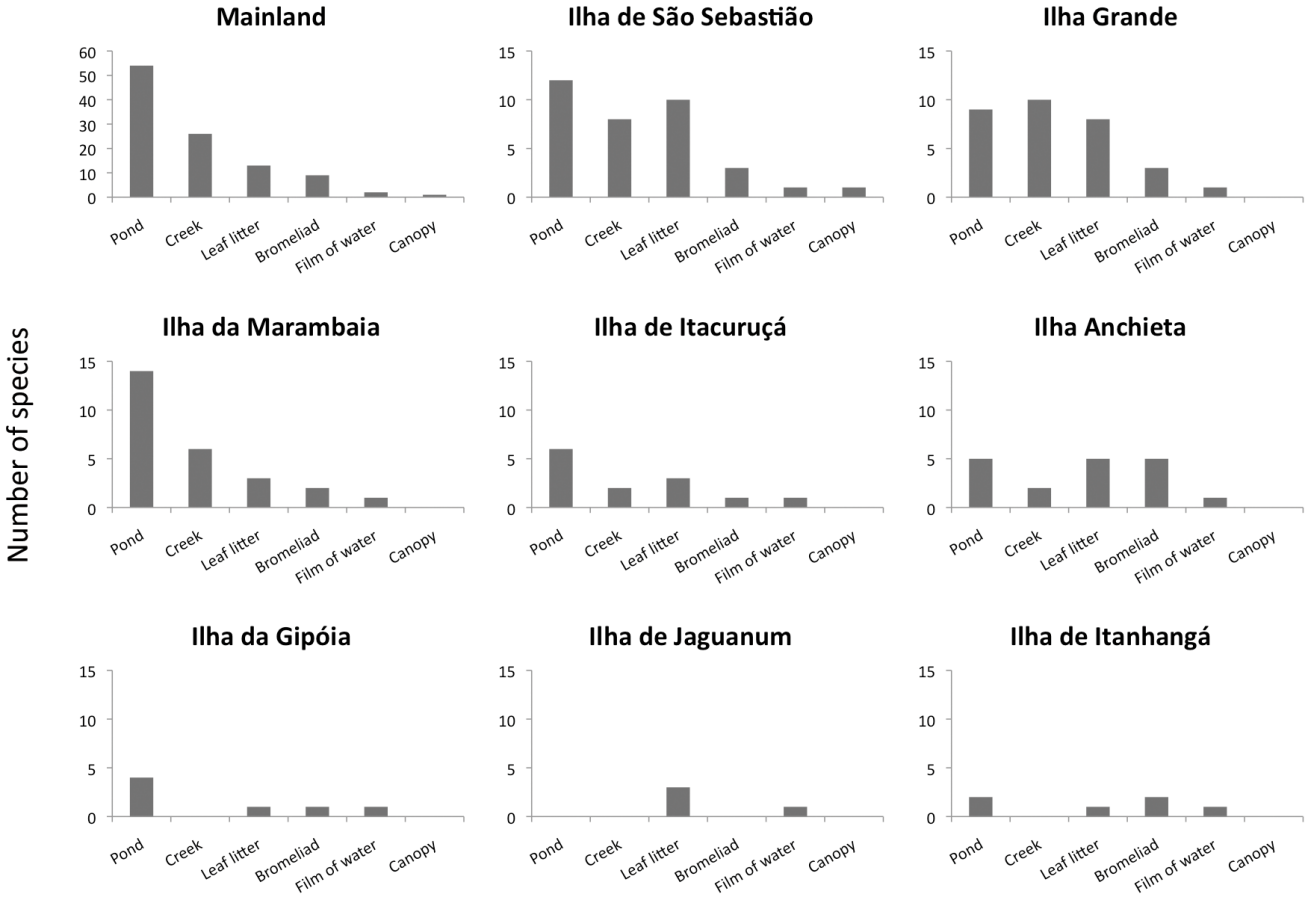
Use of breeding habitats by anuran species, not considering the macro-habitats where they occur.

From the species records we counted 23 reproductive modes (RMs) in the mainland and a subset of 17 of these on the islands (Table S3). The most common RM used by anurans, characterized by eggs laid on lentic water bodies (M1), was the most frequent in terms of number of species amongst both mainland (36%, n = 38) and islands (27%, n = 17), followed by M23 (eggs laid on leaf litter; direct development), representing 10% (n = 10) in the mainland and 17% (n = 11) on the islands. Mode 19 (eggs laid on films of water over rocks) and M32 (foam nest in subterranean constructed chambers), both describing species that are independent of water bodies for reproduction, were recorded on all islands. Ilha de Jaguanum is the only island where there is no record of modes M1 or M6 (eggs laid in bromeliads). Figure 3 shows the percentage of anuran species with RMs dependent and independent of water bodies. On Ilha de Jaguanum and Ilha de Itanhangá, the smallest islands sampled, the number of species with RMs independent of water bodies accounts for 67% and 100%, respectively. Figure 4 gives the number of species per breeding habitat for the islands and the mainland. Despite its medium size and in contrast to the other islands, Ilha da Marambaia shows the same percentage (77%) of species dependent on water bodies for reproduction as the mainland (see discussion).

## Discussion

### Species-area relationship on land-bridge islands

That no significant relationship was detected between species richness and distance of islands to the mainland confirms our expectation that the observed pattern of species richness is not explained by post-isolation colonization events. Although larger islands typically have more species than smaller ones [8], [16], the idea that habitat heterogeneity, in addition to island area, plays an important role in determining the number of species on islands seems to be well-supported by a number of studies [6], [46], [47]. Our results agree in showing that area has both a direct and an indirect effect upon the number of species, and that the indirect effect seems to be associated in a non-linear manner with the number of breeding habitats available on each island. Although area makes a stronger contribution to the number of species on the islands, the effect of NBH is also important.

Williams [48] asserts that area will only have a positive effect on species richness when positively correlated with habitat diversity. Larger islands are usually more diverse in habitats than are smaller ones. When we compare the number of species on small and very small islands (see Table 1) we notice an increment in species richness without a significant increase in area. On these islands, the presence of certain breeding habitats has a stronger effect on the species richness than does area. Additionally, on islands with comparable areas, such as Ilha de Itacuruçá and Ilha Anchieta, it is clear that despite their similar NBHs, the abundance of these habitats (i.e. number of streams and ponds, presence of ponds in different altitudes, etc) also has an impact on the number of species present on each island. Ilha Anchieta has more than ten streams, whilst Ilha de Itacuruçá has only two. The structural complexity of the first island is possibly responsible for its larger number of species, when compared to Ilha de Itacuruçá. Furthermore, the presence of certain breeding habitats (e.g. ponds and streams) can potentially amplify substantially the species diversity of an island, because there are more species that use streams than using bromeliads or films of water as breeding habitats (Table 5). Zimmerman and Bierregaard [49] studied frog communities of forest fragments in the Amazon and showed that the number of suitable breeding habitats is a better predictor of species richness than the fragment area. This similarity in patterns observed between islands and continental forest fragments suggests the existence of a general ecological process (i.e. selective extinction driven by habitat loss) responsible for regulating frog species compositions in these fragmented environments over quite different timescales.

**Table 5:** Number of anuran species per breeding habitat (BH) in each macro-habitat (MH) on eight islands off the southeastern coast of Brazil.

BH	MH	SAO	GRD	MAR	ITA	ANC	GIP	JAG	ITN	Total
Bromeliad	Forest	2	2	1		4			1	6
	Forest/Open area	1	1	1	1	1	1		1	7
Pond/TempPond	Forest	7	6	3	3	3	2		1	9
	Forest/Open area	2	2	2	2	1	1		1	4
	Open area	3	1	5	1	1	1			7
	Restinga			4						4
Canopy	Forest	1								1
Stream	Forest	8	10	6	2	2				15
Film of water	Forest/Open area	1	1	1	1	1	1	1	1	2
Leaf litter	Forest	10	8	3	3	5	1	3	1	14
**Total**		**35**	**31**	**26**	**13**	**18**	**7**	**4**	**6**	

Ilha de São Sebastião (SAO), Ilha Grande (GRD), Ilha da Marambaia (MAR), Ilha Anchieta (ANC), Ilha de Itacuruçá (ITA), Ilha da Gipóia (GIP), Ilha de Jaguanum (JAG), Ilha de Itanhangá (ITN).

### Mainland vs. islands

When compared to mainland fragments of similar sizes, insular communities usually show a significant reduction of species richness [7], [50], [51]1. A study of anurans on the Zhoushan archipelago in China shows that small islands have impoverished anuran fauna, but large islands (>100 km^2^) have similar numbers of species when compared to mainland fragments of comparable sizes [51]. These authors suggest that selective extinction played an important role in controlling species richness on islands, especially on smaller ones. Other taxa, such as mammals, birds and reptiles, also show reduction of species richness on islands [7], [52], [53]. Species impoverishment is evident when we compare the list of anurans of Estação Biológica de Boracéia (EBB), in São Paulo [54], [55] and of the Municipality of Rio de Janeiro (MRJ) [56], both with >60 species of anurans, with the species list of Ilha de São Sebastião and Ilha Grande, the two largest islands included in this study, which have 35 and 31 species, respectively. Lists of anuran fauna on mainland localities of comparable sizes to the studied islands are rare. In terms of area, EBB (165 km^2^) is comparable to Ilha Grande (193 km^2^); however, the first is part of a continuous forest, which facilitates immigration of individuals (even from new species) and maintenance of current populations. In contrast, the list of MRJ includes an area substantially larger and more diverse than any of the islands (Rio de Janeiro: 1,260 km^2^). Furthermore, the lists here used for comparison (EBB and MRJ) are the results of long-term studies. Comparisons of species richness between islands and mainland must therefore be made with caution; nevertheless, in this study our findings corroborate the general pattern of species impoverishment on islands.

When species were arranged by their reproductive habitat, we noticed that most of the lowland temporary-pond breeders, commonly found in the adjacent mainland [57], [58], were absent from the islands. Species such as *Dendropsophus anceps*, *D. bipunctatus*, *D. elegans*, *D. minutus*, *Phyllomedusa* spp., *Scinax similis* and *S. fuscomarginatus* are commonly found in the mainland, but absent on the islands. Most of them depend on breeding habitats typical of open lowland areas such as swamps and ponds. *Leptdactylus fuscus* is commonly found on open habitats on the mainland, is well adapted to human altered habitats and is so abundant that it is considered ‘weedy’ [59]. Ephemeral and permanent ponds in open areas that are used by this species as breeding habitats temporarily disappeared from the lowlands, both on islands and on the mainland, during sea-level transgressions in the past. Populations of lowland species from both islands and the mainland probably went extinct, though the mainland areas were re-colonized after the regression of the sea level. It is possible that some leaf litter and canopy-dwelling species may have been overlooked as a consequence of the different sampling methods employed on each island. In contrast, we consider it unlikely that the above-mentioned species have been overlooked. Some anuran species recorded on the adjacent mainland localities are restricted to high elevations [see references in 60] that are found only on a few islands which is an obvious constraint on the extend of their occurrence on the islands. Another possible explanation for the absence of some species on the islands is their small geographical range.

### Species composition

It is clear that species are not randomly distributed on the study islands and a nested distribution of species was already expected based on many previous studies of different groups of animals and plants [15], [51], [61]. Although many processes are known to generate nested patterns of species composition [15], [62], selective extinction is thought to be the major cause of nestedness on land-bridge islands [15], [51], [63]. The absence of several species on the islands, all dependent on temporary water bodies in open areas for reproduction and that are also commonly found in the mainland, lead us to the conclusion that the nested pattern observed resulted from a selective extinction process, driven by habitat loss. About five thousand years ago the relative sea level was 3.5±1 m above the present level, leading to marine transgressions [19], [20] that inundated the coastal plain in the mainland and the already limited lowland open areas on the islands. Swamps, marshes and ponds disappeared from these lowland areas during this period, possibly driving populations of those species that reproduce exclusively in these habitats extinct.

The similarity observed between Ilha da Marambaia and the mainland in the proportion of species dependent on water bodies for reproduction is possibly due to its connection with the mainland which may serve as a selective dispersal route for some species associated with this habitat. Characterized by a xeric environment, classified here as restinga habitat, this tombolo probably serves as a dispersal route for *Aparasphenodon brunoi*, *Xenohyla truncata* and *Rhinella pygmaea*. These species are well adapted to this habitat type and commonly found in other restingas along the coast of Rio de Janeiro [64], [65]1, avoiding water loss during the day by hiding in bromeliads (*A. brunoi* and *X. truncata*) or burying in the sand (*R. pygmaea*), and using ephemeral ponds formed after heavy rains as breeding habitats. Two other islands also have restinga habitats (see Table 3) but, unlike Ilha da Marambaia, they are not connected to the mainland and these three species are absent from them.

Given that the availability of suitable breeding habitat is a constraint on the presence of reproducing populations of anurans, it is important to note that breeding habitats are not equally distributed throughout the islands. For instance, there are no permanent water bodies on Ilha de Jaguanum and Ilha de Itanhangá and, unsurprisingly, no stream-dwelling frogs (e.g. *Hylodes* spp and *Crossodactylus* spp). In contrast, outcrops of rocks and leaf litter, and the anuran species that use these as breeding habitats (*Thoropa* spp and *Adenomera marmorata*, respectively), are present on all the islands. Similarly, bromeliads are another potential breeding habitat used by anurans on all islands and, with the exception of Ilha de Jaguanum. There are several reports of overseas dispersal in amphibians [66]–[68], however, on our study islands, species composition is essentially explained by vicariance.

### Conservation

The conservation of a high diversity of frogs in the BAF depends on the preservation of more than just large areas. The distribution pattern and loss of species diversity observed on land-bridge islands indicates that, at least for anurans, the maintenance of a high diversity of breeding habitats may be the main factor for preserving species diversity in the BAF. The protection of breeding habitats used by frogs appears to be a strong component on the maintenance of high species diversity in fragmented areas. Habitat heterogeneity could compensate the effect of area, so that small but structurally complex islands can be more efficient than larger homogeneous islands. The strong positive correlation between island area and habitat heterogeneity makes it difficult to disentangle the effects of these variables upon species richness.

A detailed study of reproductive modes and breeding habitats used by anurans and their distribution and abundance in the study area could provide a better understanding on the process of species loss. Past climatic changes that possibly caused loss of species habitats and diversity on the islands may give us an idea of what could happen to many coastal communities of frogs if the temperature of the Earth continues to rise. This information could serve as a basis for creating measures to minimise the effects of the current warming of the climate system [69].

Human occupation of the study islands is a potential threat to the local amphibian species (especially to stream-dwelling species such as *Crossodactylus* spp, *Hylodes* spp and *Cycloramphus* spp) that needs careful management. Almeida-Gomes et al [70] point out how vulnerable these species are because of their high habitat specificity so that disturbances such as removal of riparian forest and pollution could lead to their immediate disappearance.

## Supporting Information

Table S1 **List of anuran species recorded on eight land-bridge islands and adjacent localities on the mainland of the southeastern coast of Brazil.** Reproductive mode (RM) *sensu* Haddad & Prado (2005). Ilha de São Sebastião (SAO), Ilha Grande (GRD), Ilha da Marambaia (MAR), Ilha Anchieta (ANC), Ilha de Itacuruçá (ITA), Ilha da Gipóia (GIP), Ilha de Jaguanum (JAG), Ilha de Itanhangá (ITN).
(DOCX)

Table S2 **Source of database included in this study.** Species link (SL) captured until 14 June 2013 accessible at http://www.splink.org.br/. Unpublished data (UD).
(DOCX)

Table S3 **List of reproductive modes (RM)^1^.**
(DOCX)
