# Supplementary material for: Effects of Fragmentation and Sea-Level Changes upon Frog Communities of Land-Bridge Islands off the Southeastern Coast of Brazil
Source: PLoS One. 2014 Jul 28;9(7):e103522. doi: 10.1371/journal.pone.0103522 (PMC4113446; doi:10.1371/journal.pone.0103522)
Supplement: Table S1 — List of anuran species recorded on eight land-bridge islands and adjacent localities on the mainland of the southeastern coast of Brazil. Reproductive mode (RM) sensu Haddad & Prado (2005). Ilha de São Sebastião (SAO), Ilha Grande (GRD), Ilha da Marambaia (MAR), Ilha Anchieta (ANC), Ilha de Itacuruçá (ITA), Ilha da Gipóia (GIP), Ilha de Jaguanum (JAG), Ilha de Itanhangá (ITN). (DOCX) [file pone.0103522.s001.docx]

**Table S1. List of anuran species recorded on eight land-bridge islands and adjacent localities on the mainland of the southeastern coast of Brazil.** Reproductive mode (RM) *sensu* Haddad & Prado (2005). Ilha de São Sebastião (SAO), Ilha Grande (GRD), Ilha da Marambaia (MAR), Ilha Anchieta (ANC), Ilha de Itacuruçá (ITA), Ilha da Gipóia (GIP), Ilha de Jaguanum (JAG), Ilha de Itanhangá (ITN).

| **Family** | **Species** | **RM** | **MLD** | **SAO** | **GRD** | **MAR** | **ITA** | **ANC** | **GIP** | **JAG** | **ITN** |
| --- | --- | --- | --- | --- | --- | --- | --- | --- | --- | --- | --- |
| AROMOBATIDAE | *Allobates olfersioides* (Lutz, 1925) | 20 | 1 |  |  |  |  |  |  |  |  |
| BRACHYCEPHALIDAE | *Brachycephalus didactylus* (Izecksohn, 1971) | 23 | 1 |  | 1 |  |  |  |  |  |  |
|  | *Brachycephalus ephippium* (Spix, 1824) | 23 | 1 |  |  |  |  |  |  |  |  |
|  | *Brachycephalus hermogenesi* (Giaretta and Sawaya, 1998) | 23 | 1 |  |  |  |  |  |  |  |  |
|  | *Brachycephalus nodoterga* Miranda-Ribeiro, 1920 | 23 |  | 1 |  |  |  |  |  |  |  |
|  | *Brachycephalus vertebralis* Pombal, 2001 | 23 | 1 |  |  |  |  |  |  |  |  |
|  | *Ischnocnema bolbodactyla* (Lutz, 1925) | 23 | 1 |  |  |  |  | 1 |  |  |  |
|  | *Ischnocnema* aff. *erythromera* Heyer, 1984 | 23 |  | 1 |  |  |  |  |  |  |  |
|  | *Ischnocnema* gr. *lactea* (Miranda-Ribeiro, 1923) | 23 | 1 |  |  |  |  |  |  |  |  |
|  | *Ischnocnema guentheri* (Steindachner, 1864) | 23 | 1 | 1 | 1 |  |  |  |  |  |  |
|  | *Ischnocnema nasuta* (Lutz, 1925) | 27 | 1 |  |  |  |  |  |  |  |  |
|  | *Ischnocnema octavioi* (Bokermann, 1965) | 23 |  |  | 1 |  |  |  |  |  |  |
|  | *Ischnocnema parva* (Girard, 1853) | 23 | 1 | 1 | 1 |  |  | 1 |  |  |  |
|  | *Ischnocnema* sp. 1 (gr. *lactea* ) (Miranda-Ribeiro, 1923) | 23 |  | 1 |  |  |  |  |  |  |  |
|  | *Ischnocnema* sp. 2 (gr. *lactea*) (Miranda-Ribeiro, 1923) | 23 |  | 1 |  |  |  |  |  |  |  |
| BUFONIDAE | *Dendrophryniscus brevipollicatus* Jiménez de la Espada, 1870 | 8 | 1 | 1 | 1 | 1 |  | 1 |  |  | 1 |
|  | *Dendrophryniscus leucomystax* Izecksohn, 1968 | 1 | 1 |  |  |  |  |  |  |  |  |
|  | *Rhinella icterica* (Spix, 1824) | 1 | 1 |  | 1 |  | 1 |  |  |  |  |
|  | *Rhinella ornata* (Spix, 1824) | 2 | 1 | 1 | 1 | 1 | 1 | 1 |  |  |  |
|  | *Rhinella pygmaea* (Myers and Carvalho, 1952) | 2 |  |  |  | 1 |  |  |  |  |  |
| CENTROLENIDAE | *Vitreorana eurygnatha* (Lutz, 1925) | 1 | 1 |  | 1 | 1 |  |  |  |  |  |
|  | *Vitreorana uranoscopa* (Müller, 1924) | 21 | 1 |  |  | 1 |  |  |  |  |  |
| CERATOPHRYIDAE | *Ceratophrys aurita* (Raddi, 1823) | 1 | 1 |  |  |  |  |  |  |  |  |
| CRAUGASTORIDAE | *Haddadus binotatus* (Spix, 1824) | 23 | 1 | 1 | 1 | 1 | 1 | 1 |  | 1 |  |
| CYCLORAMPHIDAE | *Cycloramphus boraceiensis* Heyer, 1983 | 19 | 1 | 1 | 1 |  |  |  |  |  |  |
|  | *Cycloramphus dubius* (Miranda-Ribeiro, 1920) | 19 | 1 |  |  |  |  |  |  |  |  |
|  | *Cycloramphus eleutherodactylus* (Miranda-Ribeiro, 1920) | 19 | 1 | 1 |  |  |  |  |  |  |  |
|  | *Cycloramphus fuliginosus* Tschudi, 1838 | 19 | 1 |  |  |  |  |  |  |  |  |
|  | *Thoropa miliaris* (Spix, 1824) | 1 | 1 |  | 1 | 1 | 1 |  | 1 | 1 | 1 |
|  | *Thoropa taophora* (Miranda-Ribeiro, 1923) | 25 | 1 | 1 |  |  |  | 1 |  |  |  |
|  | *Zachaenus parvulus* (Girard, 1853) |  | 1 |  | 1 |  |  |  |  |  |  |
| HEMIPHRACTIDAE | *Fritziana fissilis* (Miranda Ribeiro, 1920) | 36 | 1 | 1 |  |  |  | 1 |  |  |  |
|  | *Fritziana goeldii* (Boulenger, 1895) | 36 | 1 |  |  |  |  | 1 |  |  |  |
|  | *Fritziana ohausi* (Wandolleck, 1907) | 36 | 1 |  |  |  |  | 1 |  |  |  |
|  | *Fritziana* sp. | 36 |  |  | 1 |  |  |  |  |  |  |
|  | *Gastrotheca albolineata* (Lutz and Lutz, 1939) | 37 | 1 |  |  |  |  |  |  |  |  |
|  | *Gastrotheca* sp. | 37 |  | 1 |  |  |  |  |  |  |  |
| HYLIDAE | *Aparasphenodon brunoi* Miranda-Ribeiro, 1920 | 1 | 1 |  |  | 1 |  |  |  |  |  |
|  | *Aplastodiscus albosignatus* (Lutz and Lutz, 1938) | 5 | 1 |  |  |  |  |  |  |  |  |
|  | *Aplastodiscus arildae* (Cruz and Peixoto, 1987) | 2 | 1 |  |  |  |  |  |  |  |  |
|  | *Aplastodiscus eugenioi* (Carvalho-e-Silva & Carvalho-e-Silva, 2005) | 2 | 1 | 1 | 1 | 1 | 1 |  |  |  |  |
|  | *Aplastodiscus leucopygius* (Cruz and Peixoto, 1985) | 5 | 1 |  |  |  |  |  |  |  |  |
|  | *Aplastodiscus perviridis* Lutz, 1950 | 5 | 1 |  |  |  |  |  |  |  |  |
|  | *Bokermannohyla circumdata* (Cope, 1871) | 4 | 1 |  | 1 |  |  |  |  |  |  |
|  | *Bokermannohyla hylax* (Heyer, 1985) | 4 | 1 | 1 |  |  |  |  |  |  |  |
|  | *Dendropsophus anceps* (Lutz, 1929) | 1 | 1 |  |  |  |  |  |  |  |  |
|  | *Dendropsophus berthalutzae* (Bokermann, 1962) | 24 | 1 | 1 |  |  |  |  |  |  |  |
|  | *Dendropsophus bipunctatus* (Spix, 1824) | 1 | 1 |  |  |  |  |  |  |  |  |
|  | *Dendropsophus* aff. *oliveirai* (Bokermann, 1963) | 1 |  |  |  | 1 |  |  |  |  |  |
|  | *Dendropsophus decipiens* (Lutz, 1925) | 24 | 1 |  |  | 1 |  |  |  |  |  |
|  | *Dendropsophus elegans* (Wied-Neuwied, 1824) | 1 | 1 |  |  |  |  |  |  |  |  |
|  | *Dendropsophus giesleri* (Mertens, 1950) | 1 | 1 |  |  |  |  |  |  |  |  |
|  | *Dendropsophus microps* (Peters, 1872) | 1 | 1 |  |  |  |  |  |  |  |  |
|  | *Dendropsophus minutus* (Peters, 1872) | 1 | 1 |  |  |  |  |  |  |  |  |
|  | *Dendropsophus sanborni* (Schmidt, 1944) | 1 | 1 |  |  |  |  |  |  |  |  |
|  | *Dendropsophus seniculus* (Cope, 1868) | 1 | 1 |  |  |  |  |  |  |  |  |
|  | *Hypsiboas albomarginatus* (Spix, 1824) | 1 | 1 | 1 | 1 | 1 | 1 | 1 | 1 |  | 1 |
|  | *Hypsiboas albopunctatus* (Spix, 1824) | 1 | 1 |  |  |  |  |  |  |  |  |
|  | *Hypsiboas* aff. *polytaenius* (Cope, 1870) | 2 | 1 |  |  |  |  |  |  |  |  |
|  | *Hypsiboas faber* (Wied-Neuwied, 1821) | 4 | 1 | 1 | 1 |  | 1 |  |  |  |  |
|  | *Hypsiboas pardalis* (Spix, 1824) | 4 | 1 |  |  |  |  |  |  |  |  |
|  | *Hypsiboas semilineatus* (Spix, 1824) | 2 | 1 |  |  |  |  |  |  |  |  |
|  | *Itapotihyla langsdorffii* (Duméril and Bibron, 1841) | 1 | 1 |  | 1 |  |  |  |  |  |  |
|  | *Phasmahyla cruzi* Carvalho-e-Silva, Silva, and Carvalho-e-Silva, 2009 | 25 | 1 |  |  |  |  |  |  |  |  |
|  | *Phasmahyla guttata* (Lutz, 1924) | 25 | 1 | 1 | 1 |  |  |  |  |  |  |
|  | *Phrynomedusa marginata* (Izecksohn and Cruz, 1976) | 18 | 1 |  |  |  |  |  |  |  |  |
|  | *Phyllomedusa burmeisteri* Boulenger, 1882 | 24 | 1 |  |  |  |  |  |  |  |  |
|  | *Phyllomedusa rohdei* Mertens, 1926 | 24 | 1 |  |  |  |  |  |  |  |  |
|  | *Scinax albicans* (Bokermann, 1967) | 1 | 1 |  |  |  |  |  |  |  |  |
|  | *Scinax alter* (Lutz, 1973) | 1 | 1 |  |  | 1 |  |  |  |  |  |
|  | *Scinax angrensis* (Lutz, 1973) | 1 | 1 |  |  |  |  |  |  |  |  |
|  | *Scinax argyreornatus* (Miranda-Ribeiro, 1926) | 1 | 1 | 1 |  |  |  |  |  |  |  |
|  | *Scinax* aff. *catharinae* (Boulenger, 1888) | 1 |  | 1 |  |  |  |  |  |  |  |
|  | *Scinax cuspidatus* (Lutz, 1925) | 1 | 1 |  |  | 1 |  |  |  |  |  |
|  | *Scinax eurydice* (Bokermann, 1968) | 1 | 1 |  |  |  |  |  |  |  |  |
|  | *Scinax fuscomarginatus* (Lutz, 1925) | 6 | 1 |  |  |  |  |  |  |  |  |
|  | *Scinax fuscovarius* (Lutz, 1925) | 1 | 1 | 1 | 1 |  |  |  |  |  |  |
|  | *Scinax* gr. *perpusillus* | 1 | 1 | 1 | 1 | 1 | 1 | 1 |  |  | 1 |
|  | *Scinax hayii* (Barbour, 1909) | 1 | 1 | 1 | 1 |  |  | 1 | 1 |  |  |
|  | *Scinax humilis* (A. Lutz and B. Lutz, 1954) | 1 | 1 |  |  |  |  |  |  |  |  |
|  | *Scinax imbegue* Nunes, Kwet, and Pombal, 2012 | 6 | 1 |  |  |  |  |  |  |  |  |
|  | *Scinax littoralis* (Pombal and Gordo, 1991) | 1 | 1 |  |  |  |  |  |  |  |  |
|  | *Scinax perpusillus* (Lutz and Lutz, 1939) | 1 | 1 |  |  |  |  |  |  |  |  |
|  | *Scinax similis* (Cochran, 1952) | 1 | 1 |  |  |  |  |  |  |  |  |
|  | *Scinax squalirostris* (Lutz, 1925) | 2 | 1 |  |  |  |  |  |  |  |  |
|  | *Scinax trapicheiroi* (A. Lutz and B. Lutz, 1954) | 1 | 1 |  | 1 | 1 | 1 |  |  |  |  |
|  | *Scinax tupinamba* Silva and Alves-Silva, 2008 | 1 | 1 |  |  |  |  |  | 1 |  |  |
|  | *Scinax tymbamirim* Nunes, Kwet, and Pombal, 2012 | 6 | 1 |  |  |  |  |  |  |  |  |
|  | *Scinax x-signatus* (Spix, 1824) | 19 | 1 |  |  | 1 |  |  |  |  |  |
|  | *Trachycephalus mesophaeus* (Hensel, 1867) | 25 | 1 | 1 |  |  |  |  | 1 |  | 1 |
|  | *Xenohyla truncata* (Izecksohn, 1959) |  |  |  |  | 1 |  |  |  |  |  |
| HYLODIDAE | *Crossodactylus gaudichaudii* Duméril and Bibron, 1841 | 3 |  |  | 1 | 1 |  |  |  |  |  |
|  | *Hylodes asper* (Müller, 1924) | 3 | 1 | 1 | 1 |  |  | 1 |  |  |  |
|  | *Hylodes fredi* Canedo and Pombal, 2007 | 3 |  |  | 1 |  |  |  |  |  |  |
|  | *Hylodes phyllodes* Heyer and Cocroft, 1986 | 3 | 1 | 1 |  | 1 |  | 1 |  |  |  |
|  | *Megaelosia goeldii* (Baumann, 1912) | 3 | 1 |  |  |  |  |  |  |  |  |
| LEPTODACTYLIDAE | *Adenomera marmorata* (Steindachner, 1867) | 32 | 1 | 1 | 1 | 1 | 1 | 1 | 1 | 1 | 1 |
|  | *Leptodactylus fuscus* (Schneider, 1799) | 30 | 1 |  |  |  |  |  |  |  |  |
|  | *Leptodactylus labyrinthicus* (Spix, 1824) | 30 | 1 |  |  |  |  |  |  |  |  |
|  | *Leptodactylus latrans* (Linnaeus, 1758) | 11 | 1 | 1 |  | 1 | 1 | 1 | 1 |  |  |
|  | *Leptodactylus marambaiae* Izecksohn, 1976 | 30 |  |  |  | 1 |  |  |  |  |  |
|  | *Leptodactylus spixi* Heyer, 1983 | 30 | 1 |  |  |  |  |  |  |  |  |
|  | *Physalaemus angrensis* Weber, Gonzaga, and Carvalho-e-Silva, 2006 | 11 | 1 |  |  |  |  |  |  |  |  |
|  | *Physalaemus atlanticus* Haddad and Sazima, 2004 | 11 | 1 |  |  |  |  |  |  |  |  |
|  | *Physalaemus cuvieri* Fitzinger, 1826 | 11 | 1 |  |  |  |  |  |  |  |  |
|  | *Physalaemus maculiventris* (Lutz, 1925) | 11 | 1 |  |  |  |  |  |  |  |  |
|  | *Physalaemus moreirae* (Miranda-Ribeiro, 1937) | 11 | 1 | 1 |  |  |  |  |  |  |  |
|  | *Physalaemus olfersii* (Lichtenstein and Martens, 1856) | 11 | 1 |  |  |  |  |  |  |  |  |
|  | *Physalaemus signifer* (Girard, 1853) | 28 | 1 |  | 1 | 1 |  |  |  |  |  |
|  | *Physalaemus spiniger* (Miranda-Ribeiro, 1926) | 2 | 1 |  |  |  |  |  |  |  |  |
| MICROHYLIDAE | *Arcovomer passarellii* Carvalho, 1954 | 1 | 1 |  |  |  |  |  |  |  |  |
|  | *Chiasmocleis carvalhoi* Cruz, Caramaschi and Izecksohn, 1997 | 1 | 1 | 1 | 1 | 1 | 1 | 1 |  |  |  |
|  | *Chiasmocleis leucosticta* (Boulenger, 1888) | 10 | 1 |  |  |  |  |  |  |  |  |
|  | *Elachistocleis cesarii* (Miranda-Ribeiro, 1920) | 1 | 1 |  |  |  |  |  |  |  |  |
|  | *Myersiella microps* (Duméril and Bibron, 1841) | 23 | 1 | 1 | 1 | 1 | 1 | 1 |  | 1 |  |
|  | *Stereocyclops parkeri* (Wettstein, 1934) | 19 | 1 | 1 |  |  |  |  |  |  |  |
| ODONTOPHRYNIDAE | *Macrogenioglottus alipioi* Carvalho, 1946 | 1 | 1 |  |  |  |  |  |  |  |  |
|  | *Proceratophrys appendiculata* (Günther, 1873) | 2 | 1 |  |  |  |  |  |  |  |  |
|  | *Proceratophrys boiei* (Wied-Neuwied, 1825) | 1 | 1 | 1 |  |  |  |  |  |  |  |
|  | *Proceratophrys tupinamba* Prado and Pombal, 2008 | 1 |  |  | 1 |  |  |  |  |  |  |
