# Supplementary material for: Effects of Fragmentation and Sea-Level Changes upon Frog Communities of Land-Bridge Islands off the Southeastern Coast of Brazil
Source: PLoS One. 2014 Jul 28;9(7):e103522. doi: 10.1371/journal.pone.0103522 (PMC4113446; doi:10.1371/journal.pone.0103522)
Supplement: Table S2 — Source of database included in this study. Species link (SL) captured until 14 June 2013 accessible at http://www.splink.org.br/. Unpublished data (UD). (DOCX) [file pone.0103522.s002.docx]

**Table S2. Source of database included in this study.** Species link (SL) captured until 14 June 2013 accessible at <http://www.splink.org.br/>. Unpublished data (UD).

| **Site** | **Source** | **Reference** |
| --- | --- | --- |
| Ilha Anchieta | Article; UD | Cicchi *et al*. 2009; Cicchi 2011 |
| Ilha da Gipóia | Article | Bittencourt-Silva & Silva 2013 |
| Ilha Grande | Book, Article | Rocha *et al.* 2009; Bittencourt-Silva & Silva 2013 |
| Ilha de Itacuruçá | Article | Bittencourt-Silva & Silva 2013 |
| Ilha de Itanhangá | Article | Bittencourt-Silva & Silva 2013 |
| Ilha de Jaguanum | Article | Bittencourt-Silva & Silva 2013 |
| Ilha da Marambaia | Article | Silva *et al*. 2008 |
| Ilha de São Sebastião | UD; SL | Sawaya 1999; Centeno 2008 |
| Angra dos Reis | SL |  |
| Caraguatatuba | SL |  |
| Mangaratiba | Article, SL | Carvalho-e-Silva *et al.* 2008 |
| Paraty | SL |  |
| São Sebastião | SL |  |
| São José do Barreiro | Article | Serafim *et al*. 2008 |
| Ubatuba | SL |  |
