# Supplementary material for: Effects of Fragmentation and Sea-Level Changes upon Frog Communities of Land-Bridge Islands off the Southeastern Coast of Brazil
Source: PLoS One. 2014 Jul 28;9(7):e103522. doi: 10.1371/journal.pone.0103522 (PMC4113446; doi:10.1371/journal.pone.0103522)
Supplement: Table S3 — List of reproductive modes (RM)1. (DOCX) [file pone.0103522.s003.docx]

**Table S3. List of reproductive modes (RM) ^1^.**

| **RM** | **Egg deposition** | **Brief description of the RM** | **Species on islands (n)** | **%** | **Species on mainland (n)** | **%** |
| --- | --- | --- | --- | --- | --- | --- |
| 1 | Egg deposited in water | Eggs and tadpoles in lentic water | 17 | 27 | 38 | 36 |
| 2 |  | Eggs and tadpoles in lotic water | 5 | 8 | 7 | 7 |
| 3 |  | Eggs and early larval stages in chambers; tadpoles in streams | 4 | 6 | 3 | 3 |
| 4 |  | Eggs and early larval stages in basins; tadpoles in ponds or streams | 3 | 5 | 4 | 4 |
| 5 |  | Eggs and early larval stages in subterranean nests; tadpoles in ponds or streams | - | - | 3 |  |
| * 6 |  | Eggs and exotrophic tadpoles in water in tree holes or aerial plants | 2 | 3 | 3 | 3 |
| * 8 |  | Eggs and endotrophic tadpoles in water in tree holes or aerial plants | 1 | 2 | 1 | 1 |
| 10 | Eggs in bubble nest | Bubble nest floating on pond; tadpoles in ponds | - |  | 1 |  |
| 11 | Eggs in foam nest (aquatic) | Foam nest on pond; tadpoles in ponds | 2 | 3 | 7 | 7 |
| 14 |  | Foam nest on water on the axils of terrestrial bromeliads; tadpoles in ponds | - | - | 1 |  |
| 18 | Eggs on ground, rocks or burrows | Eggs on ground or rock above water; upon hatching, tadpoles move to water | - | - | 1 |  |
| *19 |  | Eggs on rock or tree roots above water; semi-terrestrial tadpoles | 4 | 6 | 6 | 6 |
| 20 |  | Eggs hatch into tadpoles that are carried to water by adult | - | - | 1 |  |
| *21 |  | Eggs hatch into tadpoles that complete their development in the nest | 1 | 2 | 1 | 1 |
| *23 |  | Direct development of terrestrial eggs | 11 | 17 | 10 | 10 |
| 24 | Arboreal eggs | Eggs hatch into tadpoles that drop in lentic water | 2 | 3 | 4 | 4 |
| 25 |  | Eggs hatch into tadpoles that drop in lotic water | 3 | 5 | 4 | 4 |
| 27 |  | Eggs hatch into froglets | - | - | 1 |  |
| 28 | Eggs in foam nest (non aquatic) | Foam nest on the humid forest floor; tadpoles in ponds | 1 | 2 | 1 | 1 |
| 30 |  | Foam nest with eggs and early larval stages in nests; tadpoles in ponds | 1 | 2 | 3 | 3 |
| *32 |  | Foam nest in subterranean nests; tadpoles complete development in nest | 1 | 2 | 1 | 1 |
| *36 | Eggs carried on dorsum | Eggs carried on dorsum or in dorsal pouch of female; tadpoles in bromeliads or bamboo | 4 | 6 | 3 | 3 |
| *37 |  | Eggs carried on dorsum or in dorsal pouch of female; direct development | 1 | 2 | 1 | 1 |

1 - Adapted from Haddad & Prado (2005)

(*) RMs considered in this study as independent of water bodies
